# High-Spatiotemporal and Multimodal Soft Tactile Interface with Layered Architecture for Simultaneous Structural and Thermal Perception

**DOI:** 10.1007/s40820-026-02336-z

**Published:** 2026-09-21

**Authors:** Jaehwan Jang, Seong-Min Im, Byeong-Sun Park, Jiwon Choi, Dokyung Kim, Min-gu Kim

**Affiliations:** 1 Department of Medical Engineering, College of Medicine, Yonsei University, Seoul, 03722 Republic of Korea; 2 Department of Integrative Medicine, College of Medicine, Yonsei University, Seoul, 03722 Republic of Korea; 3 School of Mechanical Engineering, Yonsei University, Seoul, 03722 Republic of Korea; 4 Brain Korea 21 Project, Graduate School of Medical Science, Yonsei University College of Medicine, Seoul, 03722 Republic of Korea

**Keywords:** Multimodal sensing, Soft tactile interface, Tactile scanning, Soft pressure–temperature sensor array, Vision-tactile framework, Robot perception

## Abstract

Advanced robotic systems require tactile perception to interact reliably with objects in environments where visual information is limited by occlusion, illumination variations, and focusing issues. However, conventional tactile sensors are limited to single-stimulus recognition and face fundamental challenges in scalability and functionality, as miniaturizing sensor pixels reduces baseline signal levels and degrades the SNR. This study presents a high-spatiotemporal and multimodal soft tactile interface for simultaneous perception of structural and thermal profiles, fabricated through a layered architecture enabled by additive manufacturing. The proposed interface incorporates a 3D-stacked capacitive pressure sensor that combines buried interdigitated capacitors and a parallel-plate capacitor, and a reduced graphene oxide based temperature sensor within the same pixel area. The 3D-stacked electrode architecture enhances baseline capacitance within a miniaturized pixel footprint, and a via-free, interlayered interconnection scheme resolves wiring complexity in the temperature sensor array. Each sensing layer operates through independent transduction mechanisms, enabling the simultaneous perception of structural and thermal profiles with minimal cross-interference. The fabricated multimodal array can spatially resolve both contact geometry and localized thermal distributions with high-spatiotemporal resolution. Furthermore, by applying a tactile scanning strategy in robotic perception, the platform successfully identifies geometric features and surface thermal profiles of target objects even under vision-limited conditions. This study provides a scalable and robust foundation for multimodal tactile perception in advanced robotic manipulation and human–robot interaction.

**Supplementary Information:**

The online version contains supplementary material available at https://doi.org/10.1007/s40820-026-02336-z.

## Highlights

- A 3D-integrated multimodal soft tactile interface is fabricated via additive manufacturing for simultaneous perception of structural and thermal profiles.
- A layered architecture achieves high spatial density (integrating 200 sensors with 92.6 sensors cm^-2^), enhanced signal quality, and decoupled pathways, ensuring reliable multimodal sensing with minimal cross-interference.
- A tactile scanning strategy enables simultaneous identification of geometric features and hot-spot temperatures across a large area of target objects when visual information is limited.

**Supplementary Information:**

The online version contains supplementary material available at https://doi.org/10.1007/s40820-026-02336-z.

## Graphical abstract

**Figure:**
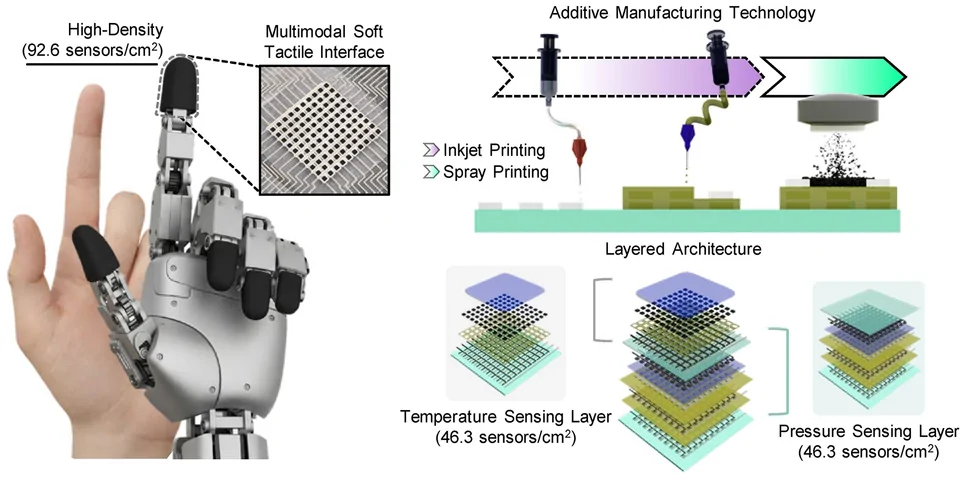

## Introduction

With the transition to the era of Physical AI, the demand for advanced human–robot interaction is rapidly increasing across various industrial sectors, including manufacturing, logistics, services, and healthcare [1–6]. Such robotic systems have evolved by adapting sensory modalities, such as vision and touch, to achieve dexterous and versatile manipulation [7–15]. In particular, tactile perception plays an essential role in robotic manipulation, as it enables physical interaction with target objects through the perception of dynamic structural and thermal profiles [16–20]. Furthermore, tactile sensing can serve as a crucial sensory substitute when visual information, such as red–green–blue (RGB) and infrared (IR), is limited by occlusion, illumination variations, and focusing issues [21, 22]. However, conventional tactile sensors are often limited to single-stimulus recognition and face challenges in scalability and functionality. This has led to an increasing demand for tactile platforms capable of detecting multiple stimuli in a high-spatiotemporal and integrated manner.

In a tactile interface, pressure sensors are a key component for quantifying contact information between the robotic hand and the target object. In particular, pressure sensor array configuration enables spatiotemporal mapping of structural profiles, such as contact location and geometric features of the object. Accordingly, recent studies have focused on increasing the sensor density to achieve high-spatial resolution pressure mapping [23–26]. However, when the sensing units are miniaturized to enhance spatial resolution, the smaller sensing area of each unit cell results in a lower output signal [27]. This low baseline signal is often not compatible with the readout integrated circuits (ICs). Furthermore, this signal reduction makes it difficult to distinguish pressure-induced signals from noise in high-density arrays, thereby degrading the signal-to-noise ratio (SNR) and ultimately deteriorating overall sensor performance. To address this issue, a materials strategy that adapts high-dielectric materials as a pressure-sensitive layer has been proposed to ensure sufficient initial signals [28–31]. However, this method still faces limitations due to the reduced electrode area being less than a millimeter scale, which affects baseline capacitance and sensing performance, thereby limiting high-density integration.

Along with spatial resolution, a multimodal tactile sensing capability is essential when a robot handles objects that are sensitive to environmental factors, such as grasping force or external heat [32–34]. Temperature sensors integrated into a robotic hand play an important role in quantifying the object's surface temperature profile and gradients. In human–robot interactions, particularly with collaborative robots, temperature information is crucial for ensuring safe engagement [35, 36]. In an industrial setting, temperature sensing enables real-time identification of areas with concentrated heat and early detection of abnormal heating. To realize a multimodal tactile interface that integrates temperature and pressure sensing, a decoupling strategy is essential for simultaneously acquiring mechanical contact and thermal information while minimizing cross-interference between mechanical and thermal stimuli. To address this issue, both homogeneous and heterogeneous multimodal sensing strategies and mechanisms have been explored [37–45]. Homogeneous approaches based on the same transduction mechanism can simplify system configuration by employing a common readout scheme. However, due to crosstalk signals from various stimuli, additional calibration procedures, compensation logic, or signal-decoupling algorithms are often necessary. In contrast, heterogeneous sensing mechanisms provide physically distinct signal pathways, enabling direct discrimination of stimuli. Nevertheless, implementing heterogeneous multimodal sensing in a high-density array remains challenging, particularly because resistive-type temperature sensors require complex wiring and interconnections in planar array configurations. Although individual wiring for each pixel can be used, this approach increases the wiring area and reduces spatial resolution [46–49]. A geometric modification incorporating a sandwich structure with vertical channels has also been proposed, but these structures suffer from reduced measurement accuracy due to their increased thickness and undesired heat transfer through the upper substrate [50, 51]. Mechanically and electrically hybrid network-based approaches have also demonstrated decoupled pressure and temperature sensing by regulating mechanical deformation and electrical transport pathways within functional material networks [52–54]. However, scalability toward high-density array integration remains challenging.

**Fig. 1:**
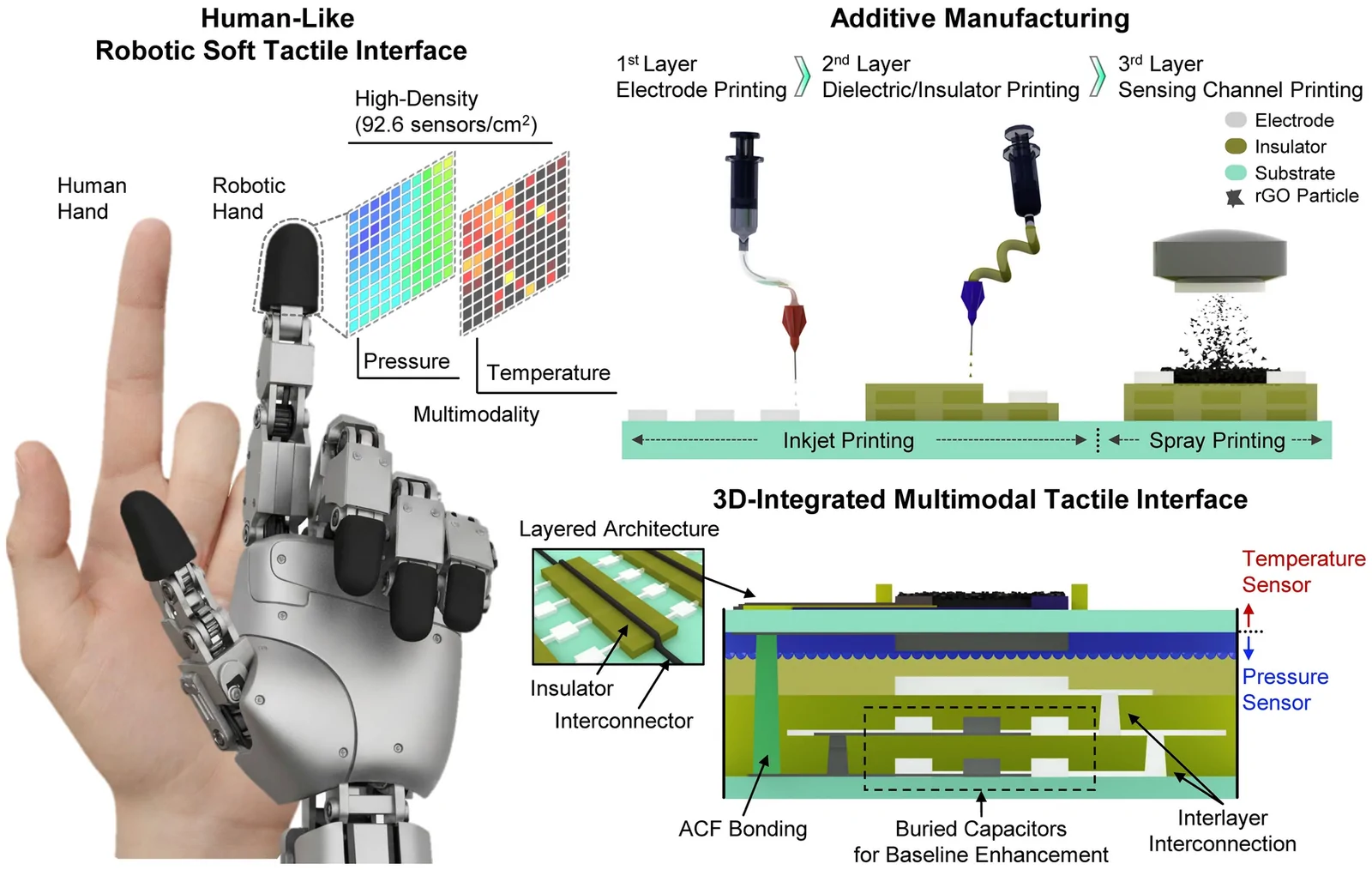
Multimodal robotic tactile perception through an additively manufactured 3D-integrated pressure–temperature sensor

In this study, we propose a high-spatiotemporal and multimodal soft tactile interface enabled by a layered architecture through additive manufacturing for simultaneous perception of shape and thermal profiles, as illustrated in Fig. 1. To address the limitations in scalability and functionality, both the pressure and temperature sensor arrays are designed with a pixel pitch of approximately 750 μm × 750 μm and manufactured using soft electronic materials via automated printing processes. These sensor arrays form a layered architecture to achieve high-spatial resolution (incorporating a total of 200 sensors with a density of 92.6 sensors cm^−2^) and multimodality for simultaneous perception of structural and thermal profiles with minimal cross-interference. The proposed architecture is designed to address challenges associated with high-density multimodal tactile interfaces, including signal degradation in miniaturized capacitive arrays, wiring complexity in temperature sensor arrays, and multimodal integration within a mechanically compliant platform. Specifically, a 3D-stacked pressure-sensing architecture that combines buried interdigitated capacitors and parallel-plate capacitors is fabricated via an inkjet-printing process using soft electronic materials. By incorporating buried interdigitated capacitors within the same pixel footprint, the baseline capacitance is enhanced without increasing the sensing area, thereby mitigating signal degradation and supporting reliable signal discrimination in high-density sensor arrays. For the temperature sensors, a resistance temperature detector (RTD) using reduced graphene oxide (rGO) as the sensing channel is fabricated via a hybrid inkjet/spray-printing process. The temperature sensors were expanded into an array at the same pixel density using a via-free and interlayered interconnection, enabling wide-range and dynamic temperature sensing. Finally, the pressure and temperature sensor arrays are vertically stacked into a 3D layered architecture, enabling simultaneous perception of mechanical and thermal stimuli with minimal cross-interference. The use of distinct capacitive and resistive sensing mechanisms facilitates stable multimodal operation in high-density arrays. A detailed comparison of previously reported pressure, temperature, and multimodal tactile sensors is presented in Table S1. This emphasizes the beneficial features of the proposed platform, including enhanced pressure sensing, scalable temperature array interconnection, high-density multimodal integration, and mechanical compliance.

## Experimental Section

### Materials

Soft conductive ink based on silver nanoparticles (AgNP, Sink01NP) was purchased from Nanopaint and used as received. Soft insulator ink (SI3104) was purchased from ACI Materials and used as received. Styrene–ethylene–butylene–styrene (SEBS H1062 and H1221) was purchased from Asahi Kasei. Sylgard 184 and its curing agent were purchased from Dow Corning. rGO powder (High Porosity Reduced Graphene Oxide) and multi-walled carbon nanotube (MWCNT) powder were purchased from Graphene Supermarket. Trichloro(1H,1H,2H,2H-perfluorooctyl)silane (PFOCTS) was purchased from Sigma-Aldrich and used as received. Poly(3,4-ethylenedioxythiophene):poly(styrenesulfonate) (PEDOT:PSS, Clevios PH1000) was purchased from Heraeus.

### Fabrication of Multimodal Soft Tactile Sensor

#### Fabrication of Pressure Sensor

To facilitate the easy release of the soft substrate from the glass without damaging the fabricated electrodes, the glass substrate was hydrophobically modified by vapor-phase deposition of trichlorosilane in a vacuum desiccator with 0.5 mL of silane at − 0.1 MPa for 24 h [55, 56]. The soft substrate was prepared by drop-casting a solution of SEBS H1062 dissolved in toluene (1:10, w/w) onto the silane-treated glass and slowly evaporating the solvent for 24 h. Subsequently, electrodes were printed onto the SEBS substrate, and an insulating layer was printed over the electrode region using the direct-write inkjet printer (Voltera, Nova). This process was repeated twice to form the embedded capacitor layers, and the pressure-sensing electrode was then inkjet-printed onto the insulating layer. After each printing step, the samples were cured at 120 °C for 20 min to remove residual solvent and improve adhesion between the printed layers and the substrate. For the micropatterned thin film fabrication, a solution of SEBS H1062 was spin-coated onto a microdome mold at 1000 r min^–1^ and cured at 120 °C for 5 min. Subsequently, a SEBS H1221 solution (1:10 in toluene, w/w) was spin-coated onto the H1062 layer to form a bilayer film. The micropatterned thin film was then transferred onto the pressure-sensing electrode. The SEBS H1062/H1221 bilayer structure was employed to enhance adhesion to the substrate while improving the mechanical recovery of the microdome structures.

#### Fabrication of Temperature Sensor

rGO was dispersed in isopropyl alcohol (IPA) at a concentration of 1 mg/mL. The mixture was treated using tip ultrasonication (40% amplitude, 20 min, Bandelin HD4100) to ensure uniform dispersion of the rGO flakes. Spray printing was performed in a temperature-controlled environment at 80 °C. A metal mask was attached to the substrate to expose only the AgNP electrode region, and 6 mL of the rGO/IPA solution was spray printed onto the defined channel area. For encapsulation, a mixture of PDMS base and curing agent (10:1, w/w) was evenly distributed using an air gun and subsequently cured at 100 °C for 2 h.

### Electrical & Mechanical Characterization

Force was controlled and recorded using a force gauge (Series 7, Mark-10) and a motorized stage (ESM303, Mark-10). Capacitance measurements were conducted using an LCR meter (E4980A, Keysight) at 1 V and 1 MHz. A source meter (Model 2400, Keithley) was used to drive the commercial Peltier device (TEC1-12706A) and to record electrical resistance. All instruments were controlled and synchronized through LabVIEW (National Instruments) as required for each measurement condition. Precise temperature control was achieved using a hot chuck controller (ST1, MSTECH). The height and surface roughness (*R*_a_) of the microdome and rGO channel were measured using a 3D analyzer (NV2400, NanoSystem). Cross-sectional photographs were obtained using an optical microscope (BX53, Olympus).

### Simulation & Robot Operation

Finite element analysis (FEA) was conducted using COMSOL Multiphysics (v6.3). The electromechanical and heat transfer physics module was employed to analyze electromechanical/thermal characteristics of pressure and temperature sensors. A four-fingered robotic platform (BlueRobin Hand, BlueRobin Inc.) equipped with current-controlled actuators at each joint was operated within a Linux-based robot operating system (ROS) framework. During tactile scanning, the contact force was controlled by regulating the motor current (− 50–50 mA), and the scanning speed was set to 80 mm/s. The robotic fingertip repeatedly contacted and released the object while the target position was shifted by a predefined distance for sequential profile acquisition, with a waiting period of approximately 5–7 s after each contact to account for the different response and recovery characteristics of the pressure and temperature sensors.

### System Configuration

An anisotropic conductive film (ACF) (Z-axis conductive tape, 3 M) was attached to the contact pads of the tactile sensor array, and a flexible flat cable (FFC) was used to connect the sensor to the readout system. A multiplexer (CD74HC4067, SparkFun) and a microcontroller unit (MCU) (Arduino MEGA2560) were used to sequentially read the capacitance signals from each electrode in the sensor array. The processed signals were visualized in real time using MATLAB. To establish a stable baseline after sensor integration, the initial capacitance was calibrated and subsequent measurements were processed in real time as ΔC/C_0_. RGB and IR images were captured using an RGB camera (MeetUp 2, Logitech) and IR camera (AT61, Raythink).

### Statistical Analysis

In the initial capacitance comparison plots for different electrode configurations, the mean and standard deviation (SD) were calculated from five samples for each electrode type (*N* = *5*). The mean and SD of the resistance change under humidity conditions and during heating and cooling were calculated from three samples (*N* = *3*), and the coefficient of determination (R^2^) was obtained using linear regression analysis. For the decoupling analysis, the mean and SD of the pressure and temperature sensor responses under pressure, thermal, and combined stimuli were calculated from five samples (*N* = *5)*. For the baseline signal analysis of the pressure and temperature sensor arrays, batch-to-batch and pixel-to-pixel variations were evaluated using six independently fabricated arrays (*N* = *6*). For the cross-interference analysis, the mean and SD of the crosstalk were calculated from four to five measurements for each neighboring region (*N* = *4−5*). For the mechanical hysteresis and linearity analyses, the pressure sensor was subjected to 50 repeated loading/unloading cycles up to 80 kPa, and the mean and SD of the capacitance response were calculated at each pressure interval for both loading and unloading processes (*N* = *50*).

## Results and Discussion

### 3D-Stacked Soft Capacitive Pressure Sensor with Enhanced Baseline Capacitance

Pressure sensors have been developed based on various sensing mechanisms, including piezoelectric, resistive, and capacitive principles [57–66]. Among these, capacitive pressure sensors offer advantages, such as low power consumption, structural stability, and minimal signal drift. In terms of scalability, the baseline capacitance corresponds to the size of the sensing electrode. Thus, when the electrode area is reduced to achieve high-spatial resolution arrays, the baseline capacitance decreases, thereby degrading the signal-to-noise ratio.

**Fig. 2:**
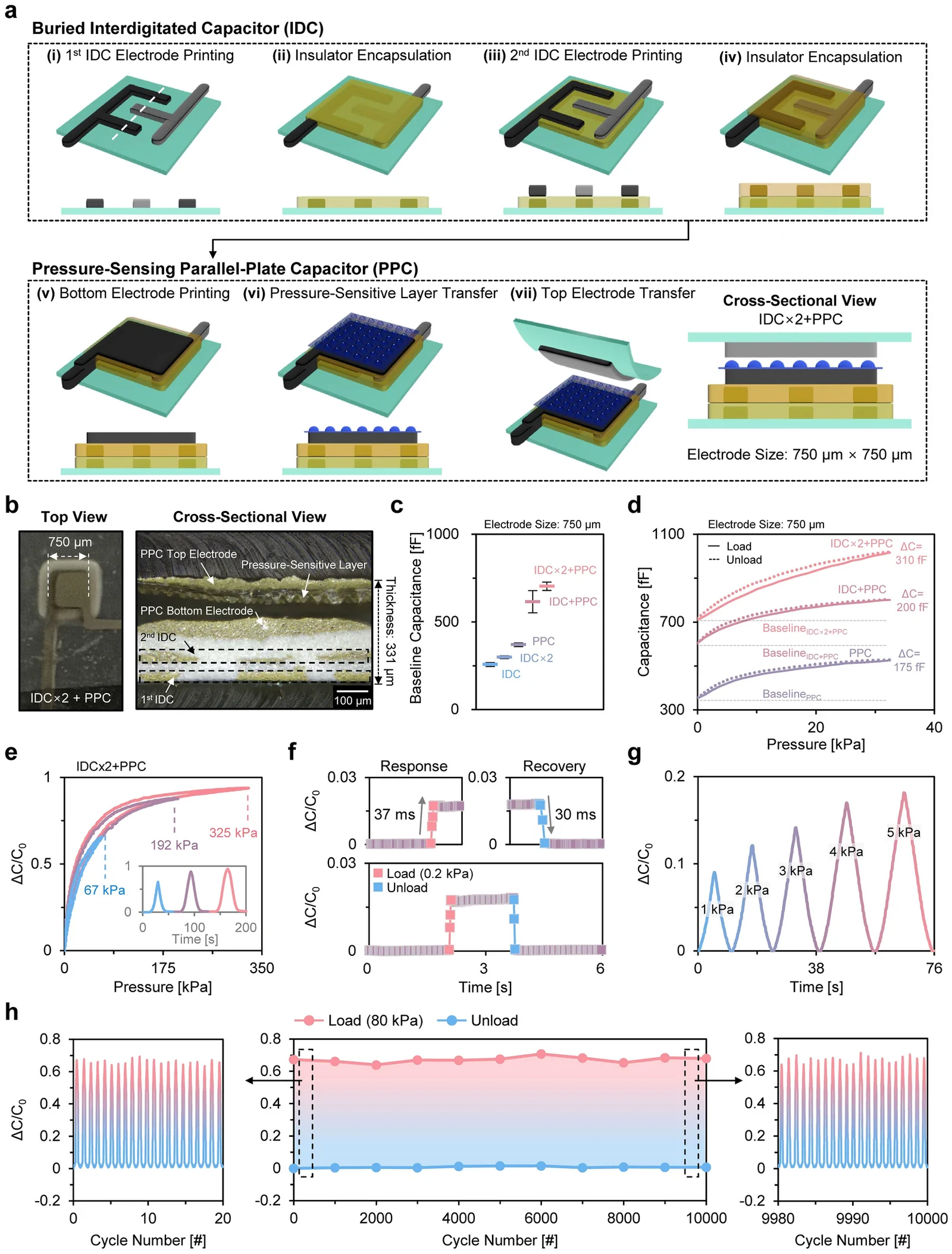
Design and electromechanical characterization of stacked soft capacitive pressure sensor. **a** Fabrication process and overall configuration of the stacked soft pressure sensor. **b** Top-view and cross-sectional photographs of the stacked soft pressure sensor architecture. **c**, **d** Electrical characteristics of each sensor with different configurations. **c** Comparison of initial capacitance (*N* = *5*). **d** Pressure-dependent capacitance variation. **e** Pressure-sensing performance under high-pressure conditions (Inset: stepwise response and recovery characteristics). **f** Dynamic pressure response and recovery behaviors. **g** Time-dependent relative capacitance changes under stepwise loading pressure of 1 kPa. **h** Electromechanical cyclic test (80 kPa, 10 k cycles)

Figure 2a illustrates the fabrication process and structure of the 3D-stacked soft capacitive pressure sensor. The sensor was fabricated using soft AgNP-based conductive inks and soft insulating inks on a soft elastomeric substrate via automated inkjet printing (Nova, Voltera Inc.). The automated inkjet-printing process allows for high-spatial resolution patterning over large areas without the need for microfabrication in a cleanroom environment [67]. It also provides precise control over electrode thickness and geometry, enabling the formation of stacked electrode structures. To overcome the reduction in baseline capacitance, a 3D parallel-stacked capacitor configuration was adopted to increase it while maintaining the same unit area. Specifically, three capacitors were connected in parallel to increase the baseline capacitance. To enhance capacitance while minimizing the overall increase in thickness, a planar interdigitated capacitor (IDC) was embedded at the bottom as a baseline capacitance-enhancement layer. To prevent electrical short circuits between IDCs, one electrode was first formed, followed by stacking the insulator layer and then printing the second electrode. On top of the buried IDCs, a parallel-plate capacitor (PPC) was stacked as the pressure-sensing layer. A pressure-sensitive microdome layer (height: 19.32 ± 0.31 μm, pitch: 42.7 ± 1.2 µm) was adapted between the electrodes of the PPC to maximize the pressure-induced variation in capacitance (Fig. S1). The final structure consists of a 3D-stacked configuration in which two IDC and one PPC (IDC × 2 + PPC) are connected in parallel. Figure 2b shows the top view and cross-sectional images of the fabricated stacked soft pressure sensor. Two embedded IDC structures are formed at the bottom, serving as buried capacitors to enhance the baseline capacitance, and a PPC structure is stacked on top as the pressure-sensing unit, resulting in a total thickness of approximately 331 μm. The baseline capacitance was evaluated through both numerical simulations and experiments, as shown in Figs. 2c and S2. A PPC structure with an electrode size of 750 μm × 750 μm exhibited an initial baseline capacitance of approximately 369 fF, whereas the IDC × 2 + PPC structure increased the baseline capacitance to 703 fF in the same area. In addition, the single IDC structure and the IDC + PPC structure exhibited initial capacitances of approximately 256 and 615 fF, respectively. These results indicate that the baseline capacitance can be designed by adjusting the number of embedded IDC layers.

Figure 2d compares the pressure-dependent capacitance variation for different design configurations. Among the PPC, IDC + PPC, and IDC × 2 + PPC structures, the IDC × 2 + PPC configuration exhibited the largest capacitance change under applied pressure. This enhancement is attributed to the combined effects of deformation-induced geometric changes in the soft dielectric layer and the embedded IDC electrodes, as well as the pressure-sensitive microdome-based PPC sensing layer. To validate this result, a finite element method (FEM) simulation was conducted for the PPC-only and IDC-embedded PPC structures (Fig. S3 and Note S1). The simulated initial capacitances for both PPC-only and IDC-embedded PPC structures agreed well with the experimental values. Under applied pressure in a low-pressure regime (< 1 kPa), the simulated capacitance variation was primarily governed by the deformation of the pressure-sensitive microdome dielectric layer, resulting in similar capacitance changes for both structures. In the higher-pressure regime (> 1 kPa), however, the capacitance response becomes more difficult to fully predict using the FEM model because nonlinear deformation of the soft dielectric material and device geometry can simultaneously affect the electrical response. Therefore, the enhanced response of the IDC-embedded PPC structure at higher pressures was mainly interpreted based on the experimental results (Fig. 2d). To further support this result, the pressure response of the IDC-only structure was additionally measured (Fig. S4). The IDC-only structure exhibited pressure-dependent capacitance variation due to the mechanical softness of the dielectric layer, confirming that the embedded IDC layers do not behave as completely static capacitors. These results suggest that enhanced capacitance response is associated not only with PPC deformation but also with additional electromechanical deformation and parasitic-capacitance-related effects within the embedded IDC structure. Additionally, as reported in previous studies, the sensing performance is expected to be further improved by optimizing the micropattern geometry, such as the size, density, and spacing of the microstructures [58]. The pressure-sensing characteristics under high-load conditions are shown in Fig. 2e. The IDC × 2 + PPC structure exhibited stable capacitance variation even at pressures above 300 kPa and fully recovered to its initial state upon unloading. To further quantify the pressure-sensing behavior under repeated mechanical loading, sensitivity, linearity, and hysteresis analyses were performed over 50 loading/unloading cycles up to 80 kPa (Fig. S5). The averaged capacitance response exhibited three distinct sensitivity regions, with sensitivities of 0.021 kPa^−1^ in the 0–10 kPa range (S1), 0.009 kPa^−1^ in the 10–40 kPa range (S2), and 0.004 kPa^−1^ in the 40–80 kPa range (S3). The corresponding coefficients of determination (R^2^) were 0.981, 0.985, and 0.992 for S1, S2, and S3, respectively. The loading/unloading hysteresis was calculated to be 9.82%, indicating stable and repeatable pressure-sensing behavior under cyclic mechanical loading. Figure 2f presents the dynamic response and recovery characteristics. The response and recovery times were measured at approximately 37 and 30 ms, respectively, indicating fast and stable operation. In addition, a clear change in capacitance was observed even under a small load of 0.2 kPa, demonstrating high pressure resolution. Figure 2g shows the capacitance response as the applied pressure was increased in 1 kPa increments. The sensor clearly distinguished each pressure level, exhibiting behavior analogous to human tactile perception of incremental force changes. Finally, Fig. 2h presents the electromechanical stability of the pressure sensor under 10,000 loading/unloading cycles at 80 kPa. The standard deviations of the capacitance response at the unloaded and loaded states were only 0.4% and 1.6%, respectively, confirming the long-term structural reliability of the sensor under high-pressure loading. Consistent daily pressure responses over a week further confirmed its long-term operational stability (Fig. S6).

### 3D-Stacked Soft Temperature Sensor with Wide Range and Dynamic Sensing Capabilities

Temperature sensors have been developed using various sensing mechanisms, including thermistors, thermocouples, and RTDs. Thermistors primarily operate via a hopping conduction mechanism in metal-oxide materials, resulting in high temperature sensitivity [68, 69]. In contrast, thermocouples utilize the Seebeck effect generated at the junction between dissimilar pure metals and thus offer a wide measurable temperature range, particularly at elevated temperatures [70, 71]. However, the materials conventionally used in thermistors and thermocouples exhibit inherently rigid mechanical properties that limit their deformability, thereby restricting their applicability in soft electronics. To address this limitation, this study presents a soft temperature sensor using an RTD mechanism to achieve high sensitivity, linearity, and stable dynamic operation [72–76]. The operating principle of RTDs relies on the temperature-dependent variation in electrical resistance of the material. In general, materials used in RTDs exhibit distinct temperature-dependent resistance behaviors. Metallic materials such as Au and Ag typically exhibit a positive temperature coefficient (PTC), in which resistance increases with temperature [72]. In contrast, polymer materials such as poly(3,4-ethylenedioxythiophene):poly(styrenesulfonate) (PEDOT:PSS) and polyaniline (PANI), as well as carbon-based materials such as multi-walled carbon nanotubes (MWCNTs) and rGO, exhibit a negative temperature coefficient (NTC), where resistance decreases as temperature increases [73–76]. MWCNT, PEDOT:PSS, and rGO were investigated as candidate temperature-sensing materials. A comparative evaluation revealed that rGO showed the most pronounced response to temperature variations, outperforming the others in sensitivity, linearity, and recovery. Therefore, rGO was selected as the temperature-sensing material (Fig. S7 and Note S2).

**Fig. 3:**
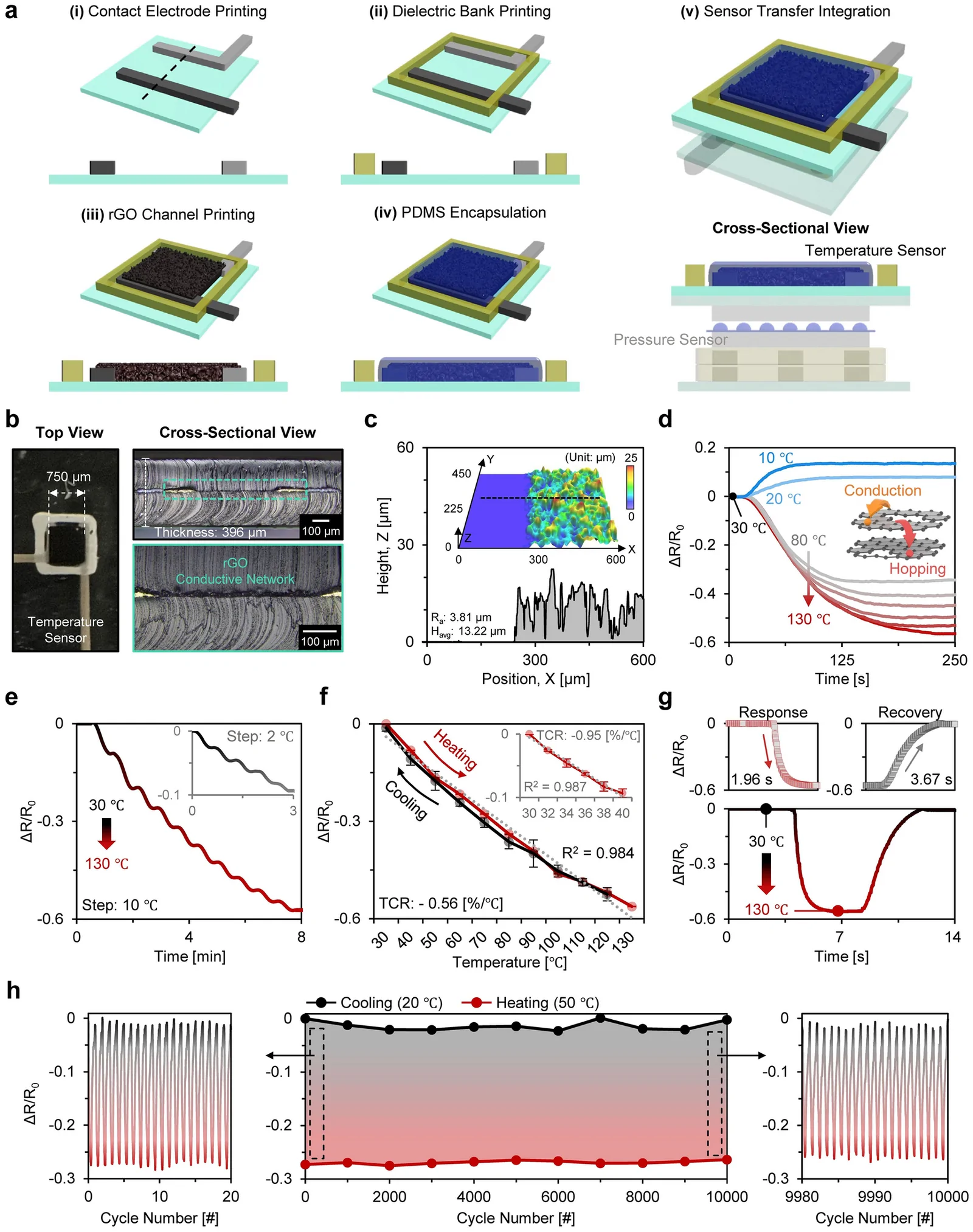
Fabrication and temperature-dependent electrical characterization of soft resistive temperature sensor. **a** Fabrication process and overall configuration of the soft temperature sensor. **b** Top-view and cross-sectional photographs of the soft temperature sensor architecture. **c** Structural characterization of rGO channel (*R*_a_: surface roughness/*H*_avg_: average height). **d** Temperature-sensing performance and working mechanism of rGO-based resistive temperature sensor. **e** Stepwise temperature sensing with 10 °C step in the range of 30–130 °C. Inset: 2 °C step in the range of 30–40 °C. **f** Statistical analysis of relative resistance changes during heating (30–130 °C) and cooling (130–30 °C) (*N* = *3*). Inset: statistical analysis of relative resistance changes in the range of 30–40 °C (*N* = *3*). **g** Dynamic thermal response and recovery behavior. **h** Temperature dependent cyclic test (23–50 °C, 10 k cycles)

The soft resistive temperature sensor was fabricated via additive manufacturing, combining inkjet printing for contact and interconnect patterning and spray printing for rGO channel formation, as illustrated in Fig. 3a. First, the contact electrodes were formed on a soft SEBS substrate by inkjet printing of a soft AgNP-based conductive ink. Subsequently, a soft insulating ink was printed to define the channel area by forming a bank structure, which was integrated with the interconnect printing process. This bank acts as a structural barrier to precisely control the channel geometry and prevent the diffusion of rGO particles during shadow-mask-assisted spray printing. Next, the temperature-sensitive layer was formed by spray printing rGO onto the defined channel region. To maintain the geometric stability of the rGO channel and minimize electrical fluctuations caused by external mechanical and chemical factors, encapsulation was performed using polydimethylsiloxane (PDMS). The temperature sensor was designed to match the dimensions of the pressure sensor. It consisted of a rGO channel with a thickness of approximately 13.2 μm and a roughness of about 3.8 μm, formed between two contacts. The entire structure was then encapsulated with polydimethylsiloxane (PDMS), resulting in a final thickness of approximately 396 μm (Fig. 3b, c).

Prior to the temperature-sensing experiments, we investigated the humidity response under different relative humidity conditions, considering the humidity-sensitive characteristic of rGO. Owing to PDMS encapsulation, the fabricated rGO-based temperature sensor showed negligible resistance variation, confirming effective suppression of humidity-induced interference (Fig. S8). Figure 3d presents the evaluation of the relative resistance and sensing range of the rGO-based temperature sensor as a function of temperature. With a baseline temperature set at 30 °C, resistance changes were recorded for target temperatures of 10 °C, 20 °C, and within a range from 80–130 °C. The results demonstrate that the signals are clearly distinguishable across a wide temperature range from 10 to 130 °C and exhibit NTC behavior, where the resistance decreased as the temperature increased, and conversely, increased as the temperature decreased. During a temperature sweep from the baseline to 130 °C in 10 °C increments, the sensor exhibited distinct resistance levels at each increment. Notably, it achieved an excellent resolution in temperature sensing as small as 2 °C (Fig. 3e). Furthermore, the sensor demonstrated reliable recovery to its initial resistance after repeated thermal cycles up to 130 °C, while exhibiting high linearity (R^2^ = 0.98) and a moderate TCR (= − 0.56% °C^–1^) in the high-temperature range (Fig. 3f). In the lower temperature range (baseline to 40 °C), superior linearity (R^2^ = 0.99) and an excellent TCR (= − 0.95% °C^–1^) were observed (Inset of Fig. 3f). To directly validate the experimental results, the electrothermal response of the rGO-based temperature sensor was further analyzed using FEM simulation (Fig. S9 and Note S3). The simulated resistance response showed a decreasing trend with increasing temperature, consistent with the experimental result, supporting the temperature-dependent sensing mechanism of the rGO channel. Additionally, rapid response and recovery times of 1.96 and 3.67 s were recorded during heating (130 °C) and cooling (30 °C) processes, respectively (Fig. 3g). These dynamic temperature response characteristics are attributed to the high thermal conductivity of rGO, which allows the sensor to rapidly reach thermal equilibrium with external temperature changes. This performance is further driven by a synergistic mechanism within the stable conduction paths of the percolation network: enhanced charge carrier mobility, a reduction in the interflake junction barrier, and increased thermally activated hopping conduction at elevated temperatures (Inset schematic of Fig. 3d). Finally, the sensor maintained its structural and electrical reliability without significant degradation over 10,000 heating and cooling cycles between 23 and 50 °C, with standard deviations of only 0.86% and 0.326% at the peak cooling and heating states, respectively (Fig. 3h). Consistent daily temperature responses over a week further confirmed its long-term operational stability (Fig. S10).

### Soft Tactile Interface for Simultaneous Structural and Thermal Profiling

**Fig. 4:**
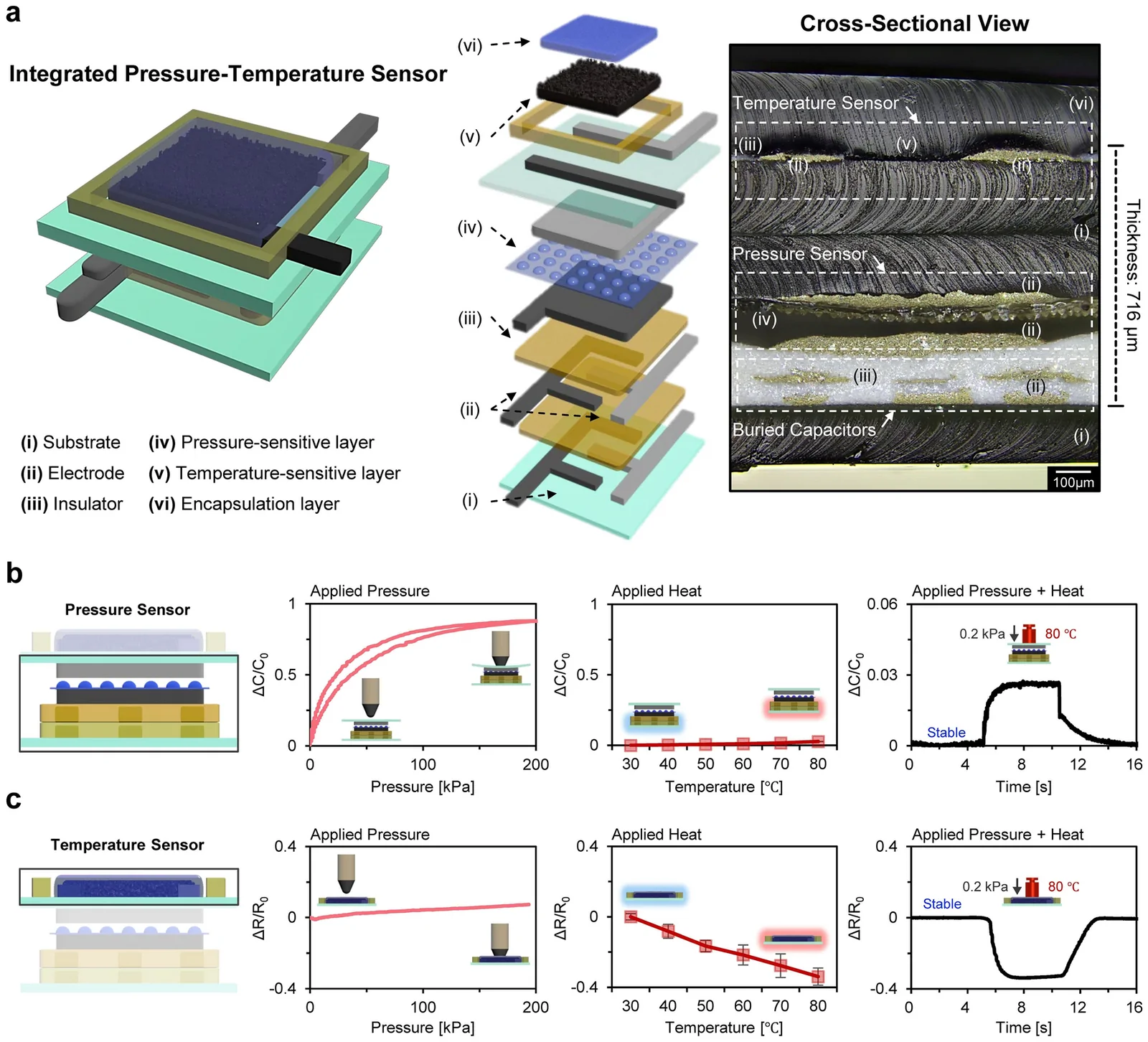
Decoupled pressure- and temperature-sensing characteristics of the integrated pressure–temperature sensor. **a** Overall configuration and cross-sectional photographs of vertically integrated pressure–temperature sensor architecture. **b** Decoupling behavior of the pressure sensor under pressure, thermal, and combined stimuli (*N* = *5*). **c** Decoupling behavior of the temperature sensor under pressure, thermal, and combined stimulus (*N* = *5*)

An integrated tactile sensor was implemented by vertically stacking a temperature sensor on a pressure sensor to validate simultaneous mechanical and thermal sensing characteristics. To minimize cross-interference during mechanical deformation and heat transfer, distinct sensing transduction mechanisms, such as capacitive pressure sensing and resistive temperature sensing, are utilized. Compared with multimodal sensors based on a single sensing mechanism, this heterogeneous sensing strategy provides intrinsically separated transduction pathways for mechanical and thermal stimuli, reducing the need for additional calibration, compensation, or signal-processing procedures [41, 42]. Figure 4a illustrates the configuration and cross-sectional images of the integrated soft tactile sensor. The underlying pressure sensor features two IDC layers to enhance baseline capacitance and a PPC layer to detect capacitance changes caused by external pressure. The upper temperature sensor is designed to detect resistance changes caused by temperature variations in the rGO channel. Both sensors were implemented on the same soft substrate with identical dimensions, resulting in a total thickness of approximately 716 µm. The cross-interference between pressure and thermal stimuli was assessed under three conditions: pressure-only, thermal-only, and combined pressure–thermal stimuli (Fig. 4b). Under pressure-only conditions, the pressure sensor exhibited an immediate and prominent change in capacitance. In contrast, a negligible signal of less than 1.5% was observed under the thermal-only stimulus condition. Under combined pressure–thermal stimuli, an instantaneous initial pressure response occurred at the moment of loading, followed by a gradual change in the signal as heat was transferred. Once the load was removed, the capacitance signal immediately decreased and subsequently returned to its stable initial state as the residual heat dissipated. These results suggest that the capacitive response of the pressure sensor remains prioritized even in the presence of thermal stimuli, demonstrating that interference from heat is limited even under high-temperature conditions at 80 °C. This characteristic was further validated through a comparative analysis with a condition where only a constant pressure (0.2 kPa) was applied (Fig. S11). Figure 4c shows the minimal cross-interference characteristics of the temperature-sensing layer in the integrated tactile sensor. While the temperature sensor exhibited distinct changes in resistance in response to thermal stimuli, negligible changes in resistance were observed under the pressure-only stimulus condition. Even in a combined stimulus environment, the temperature-driven response remained dominant, while electrical disturbances from mechanical pressure were effectively minimized. The decoupling strategy for the integrated pressure–temperature sensor is based on heterogeneous transduction mechanisms combined with material-structural design. The capacitive pressure sensor exhibits minimal interference with temperature changes, while it shows high sensitivity to external pressure through deformation of the pressure-sensitive microdome dielectric layer. In contrast, the rGO-based resistive temperature sensor shows minimal pressure-induced interference because of its 2D planar structure created by additive manufacturing and PDMS encapsulation. This design minimizes deformation of the rGO resistive pathway while allowing for a sensitive response to temperature changes. To further examine the temporal synchronization under simultaneous pressure and thermal stimuli, the response dynamics of the pressure and temperature sensors were compared under combined conditions (Figs. 4b, c and S12). Both the pressure and temperature measurements are systematically synchronized at the same sampling rate of approximately 48–50 Hz. The temperature sensor exhibited slight delays of 0.49 and 0.41 s upon loading and unloading. In addition, the temperature signal reached its peak response and returned to baseline within 1.42 and 2.01 s, respectively. This delayed thermal response is consistent with the heat-transfer-mediated nature of human thermal perception. Thus, the observed temporal difference is considered acceptable for multimodal tactile sensing, while the 3D layered architecture effectively isolates mechanical and thermal stimuli even in complex environments. The tolerance of the pressure and temperature sensors were evaluated under bending and stretching conditions. Under bending radii from 10 to 4 mm, the baseline capacitance and resistance increased as the bending radius decreased (Fig. S13). Sensing performance for both pressure and temperature were consistently maintained down to a bending radius of 4 mm (Fig. S14). Additionally, the effects of stretching deformation were evaluated by measuring the resistance change of AgNP traces under a vertically applied force. The AgNP traces demonstrated minimal resistance variation under vertical loading, suggesting that deformation induced by vertical force has a negligible effect on the electrode response (Fig. S15). Although the initial baseline signals can change under mechanical deformations, moderate deformation has only a limited effect on the pressure- and temperature-dependent sensing responses, suggesting that the sensor can operate stably in robotic environments where excessive stretching and severe bending are suppressed by the underlying finger structure. These results indicate that after the initial calibration, variations in pressure and temperature can be quantitatively monitored through relative changes in signals from the established baseline state.

The integrated pressure–temperature sensor is then scaled up into a large-area, high-spatial resolution array structure using an additive manufacturing process. One of the most critical design elements in the array expansion process is the implementation of complex interconnections while preventing short circuits between signal lines. To address this challenge, a printing-based interlayered interconnection architecture was introduced. Specifically, a multilayer wiring structure was achieved by printing an insulating ink over the bottom wiring, followed by additional printing of the top wiring (Figs. S16 and S17). This approach enables the crossing of signal lines without the need for separate via formation, effectively mitigating the wiring complexity that typically arises during array expansion.

**Fig. 5:**
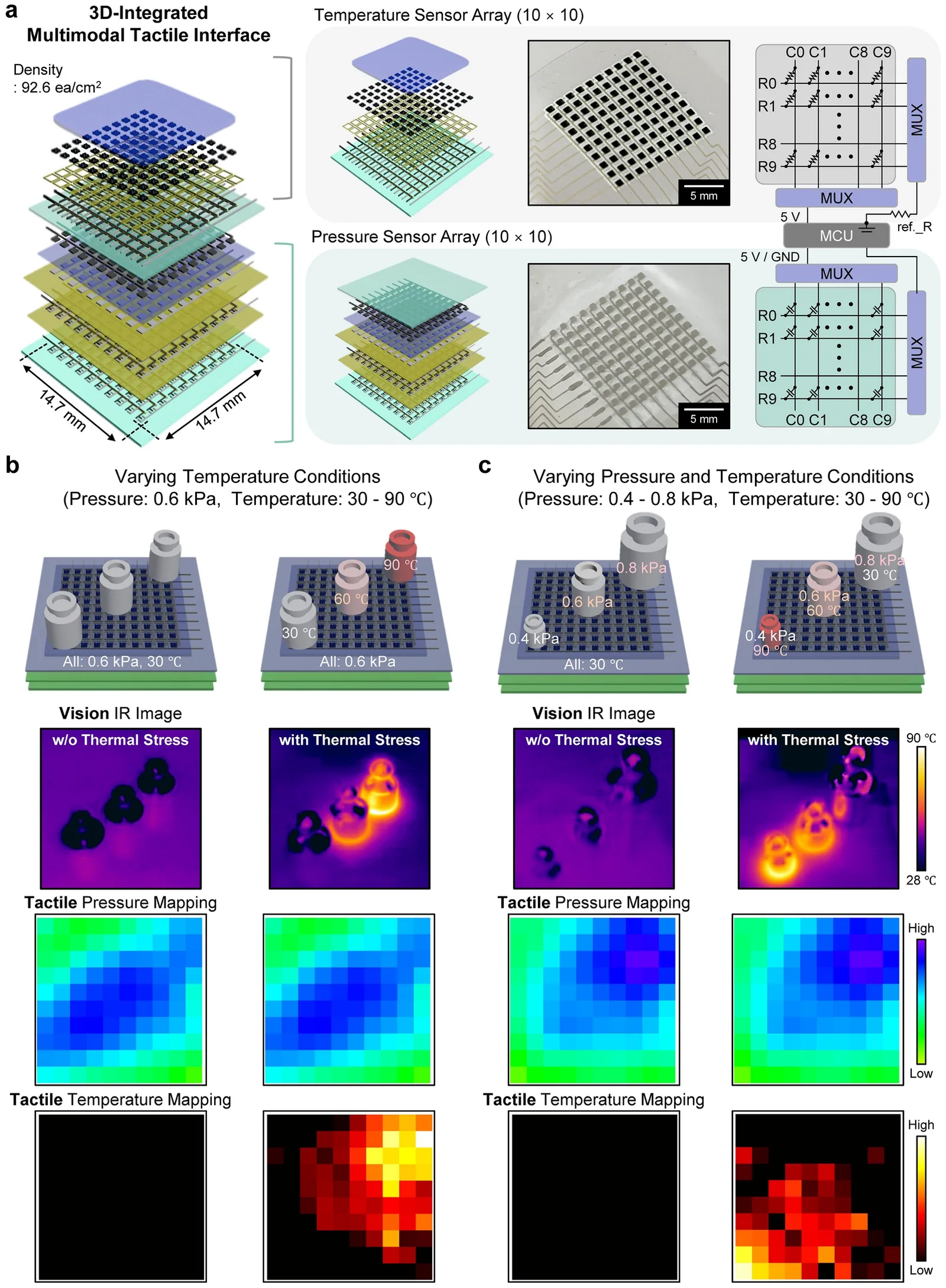
3D-integrated high-spatiotemporal and multimodal interface for simultaneous localization and temperature perception. **a** Exploded schematic and photographs of a 3D-integrated multimodal tactile sensor array with readout circuits. **b** Simultaneous localization and temperature recognition under identical pressure conditions (0.6 kPa). Left: Without thermal gradient (30 °C). Right: With spatial temperature variations (30–90 °C), demonstrating pressure-independent temperature discrimination. **c** Simultaneous localization and temperature recognition under spatially varying pressures (0.4–0.8 kPa). Left: Without thermal gradient (30 °C). Right: With spatial temperature variations (30–90 °C), demonstrating robust temperature discrimination independent of pressure variations

Figure 5a illustrates the system architecture and driving circuit of the 3D-integrated multimodal tactile interface. The fabricated multimodal tactile interface consists of a 10 × 10 pressure sensor array and a 10 × 10 temperature sensor array, which are vertically integrated and stacked on a single substrate. The array size is 14.7 mm × 14.7 mm, with a total of 200 sensor pixels integrated at a high density of 92.6 sensors cm^−2^. The array is configured as a passive matrix, designed to sequentially read signals from each pixel through row-and-column multiplexing, with the data subsequently processed by a microcontroller unit (MCU) (Fig. S18). The fabrication reproducibility of the integrated arrays was also evaluated by analyzing batch-to-batch and pixel-to-pixel variations in the baseline signals (Figs. S19 and S20). The pressure and temperature sensor arrays showed stable batch- and pixel-level responses, with batch-to-batch deviations of < 4.65% for capacitance and < 10.53% for resistance, respectively, confirming reproducible array-level fabrication through the additive manufacturing-based process. To quantitatively evaluate pixel-to-pixel crosstalk in the array, spatial interference characterization was performed (Figs. S21–S30). For the pressure sensor array, the crosstalk was calculated by defining the directly loaded pixels as the reference region with a crosstalk of 100%. The averaged crosstalk decreased with increasing distance from the loaded pixels, from 66.88 ± 8.63% in the 1st neighboring region to 9.96 ± 2.75% in the 8th neighboring region. Although residual responses were observed in nearby pixels due to mechanical coupling through the soft substrate, the distance-dependent decrease in crosstalk indicates attenuation of pressure-induced signal propagation across the array. For the temperature sensor array, the averaged crosstalk decreased with increasing distance from the stimulated pixels, from 70.14 ± 3.00% in the 1st neighboring region to 21.18 ± 3.77% in the 8th neighboring region. Although residual responses were observed in nearby pixels due to thermal diffusion from the heated region, the distance-dependent decrease in crosstalk indicates attenuation of heat-induced signal propagation across the array. These results demonstrate that both the pressure and temperature sensor arrays maintain spatially distinguishable responses despite the presence of localized mechanical and thermal crosstalk. To evaluate the spatial pressure sensing and localized temperature-variation recognition performance of the fabricated array, its sensing characteristics were analyzed under various tactile stimulus conditions. Figure 5b, c highlights the simultaneous structural and thermal profiling capabilities. In the absence of a thermal gradient (confirmed at 30 °C via an IR camera), the pressure sensor array detected the shape corresponding to the localized contact point (0.6 kPa), while no significant signal changes were observed across the entire temperature sensor array. Conversely, when spatially distinct temperatures (30, 60, and 90 °C) were applied, the temperature sensor array clearly distinguished localized temperature differences at each position, despite uniform pressure. Figure 5b demonstrates that the temperature sensors can independently detect thermal distributions without being affected by pressure, even under consistent mechanical loading. Figure 5c shows the evaluation results of the simultaneous sensing performance under spatially varying pressure conditions (0.4, 0.6, and 0.8 kPa). In the absence of a thermal gradient, the pressure sensor array clearly distinguished differences in both contact location and pressure magnitude, while the temperature sensor array exhibited no response. Conversely, even in an environment where distinct temperature distributions (30, 60, and 90 °C) coexisted, the temperature sensor array reliably identified the temperature at each position, regardless of pressure variations. To further evaluate the sensing capability, a multimodal object recognition experiment was conducted using a small wrench with curved geometric features and millimeter-scale structural details (Fig. S31). The object was preheated to approximately 80 °C prior to contact, allowing simultaneous acquisition of pressure and temperature information. The resulting pressure map successfully captured the geometric features and contact pattern of the wrench, while the temperature map clearly identified the corresponding thermal distribution. The soft and compliant characteristics of the proposed sensor allowed conformal contact with object surfaces, enabling simultaneous visualization of fine structural features and localized thermal stimuli beyond simple contact localization. Therefore, this layered architecture, enabled by additive manufacturing, minimizes cross-interference while mitigating wiring complexity and facilitating high-density multimodal sensor integration, thereby demonstrating its potential as a reliable platform for robotic tactile perception.

### Robotic Perception Based on Tactile Scanning for Large-Area Structural and Thermal Profiling

**Fig. 6:**
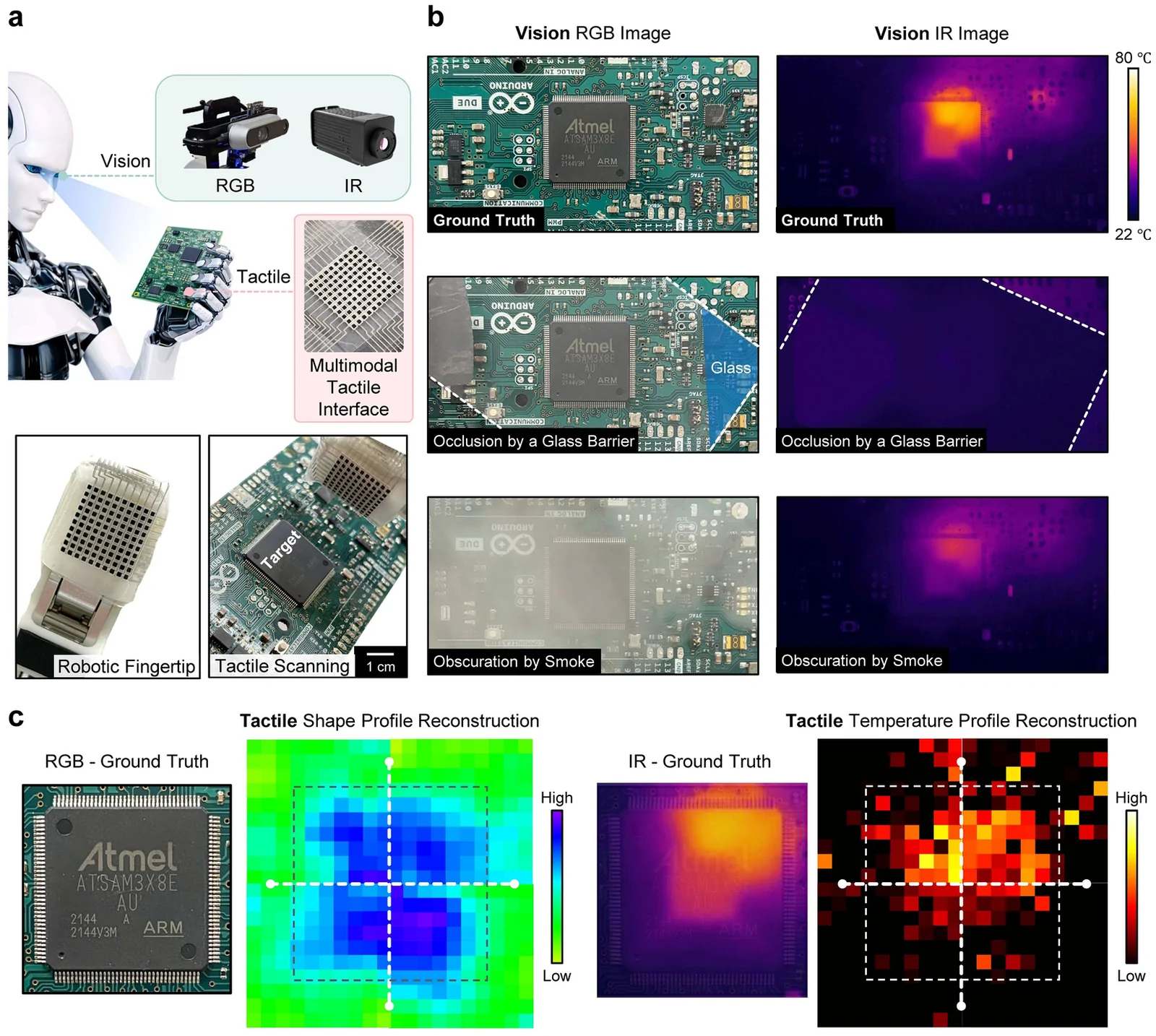
Robust tactile perception through scanning and reconstruction under visually degraded conditions. **a** Conceptual illustration of robotic multimodal tactile perception for direct object recognition. **b** Vision-based approach through RGB and IR cameras under clear and visually degraded conditions, demonstrating severe performance degradation obscured by glass and smoke. **c** Geometric feature and local thermal detection via tactile scanning technique and data reconstruction

To validate practical applicability in robotic perception, the soft tactile interface was integrated into the robotic fingertip and employed to perform tactile scanning (Fig. 6a). In general, vision-based perception relies on RGB cameras to detect object positions and shapes, whereas IR cameras are used to measure surface temperature distributions.

However, visual perception is inherently limited by external factors such as occlusion, illumination variations, defocus, and scattering by intervening media, all of which degrade the reliable estimation of geometric features, relative contact positions, and surface temperature of target objects. To demonstrate robotic perception through complementary visual and tactile modalities, a printed circuit board (PCB) incorporating a microcontroller unit (MCU, 20 mm × 20 mm) was selected as the target object, and a spatial temperature gradient was applied across the MCU. This configuration mimics localized overheating conditions commonly observed in electronic components and industrial machinery.

Figure 6b compares the RGB and IR images to capture structural and thermal profiles under normal and occlusion-occurred conditions. First, a glass layer was inserted between the camera and the object to simulate an occluded scenario. Due to the transparency of the glass, the RGB camera captured the positions and shapes of the object with reasonable clarity. However, the IR camera failed to accurately detect the temperature distribution of the object through the glass. Smoke or moisture also induces light scattering, thereby significantly reducing the contrast of RGB images and interfering with IR radiation transmission. Under these conditions, both RGB and IR cameras failed to reliably recognize the geometric features and temperature information of the target object. Multimodal tactile perception can serve as a complementary or alternative sensing modality in robotic perception when visual information is limited. Because the size of the tactile sensor is smaller than the target object, it was difficult to capture the entire surface in a single measurement. Thus, a tactile scanning strategy was adopted to reconstruct the full surface information of the object [77]. The object surface was segmented into multiple regions, sequential contact scanning was performed, and the acquired data were reconstructed to form a complete surface map (Fig. S32, Videos S1 and S2). Figure 6c presents the reconstructed results obtained through tactile scanning. By integrating data from the pressure sensor array, the geometric features of the MCU were successfully reconstructed. Simultaneously, the temperature sensor array enabled identification of the localized temperature distribution in the heated region. The measured thermal distribution showed clear spatial agreement with the ground truth obtained from IR imaging. In particular, localized temperature elevation within specific regions of the MCU was distinctly resolved. To further evaluate the robustness of the tactile scanning approach, repeated measurements were performed under identical contact conditions (Fig. S33). The reconstructed pressure and temperature maps exhibited consistent spatial distributions over multiple measurements, enabling reliable discrimination between stimulated and non-stimulated regions. These results indicate that the proposed tactile scanning strategy can reproducibly capture relative pressure and temperature variations across the object surface. These results demonstrate that multimodal tactile sensing enables direct tactile perception of object geometry and thermal distribution even in environments where visual information is limited. Furthermore, the proposed platform not only complements visual sensing but also shows strong potential as an alternative sensing modality in scenarios where visual perception fails or becomes unreliable.

## Conclusions

In this study, we proposed a high-spatiotemporal and multimodal soft tactile interface for simultaneous perception of structural and thermal profiles, enabled by a layered architecture through additive manufacturing. The proposed platform directly addressed the fundamental challenges in scalability and functionality that have limited conventional tactile sensors to single-stimulus recognition. For the pressure-sensing component, a 3D-stacked architecture combining buried IDC and PPC layers was adopted to enhance baseline capacitance within a miniaturized pixel footprint, thereby overcoming the low-signal limitation and SNR degradation inherent in high-density capacitive arrays. For the temperature-sensing component, a via-free and interlayered interconnection scheme was implemented to resolve the interconnection complexity that constrains resistive temperature sensors in planar high-density arrays, avoiding both the spatial resolution penalty of individual pixel wiring and the measurement inaccuracy associated with sandwich-type vertical-channel structures. By vertically integrating these two sensing layers into a layered architecture, the platform achieved simultaneous perception of structural and thermal profiles across 200 sensors at a density of 92.6 sensors cm^−2^. In a robotic manipulation environment, the system successfully identified both the geometric features and localized thermal distributions of target objects even under vision-limited conditions, confirming its practical effectiveness as a crucial sensory substitute. These results demonstrate that additive manufacturing-enabled layered architectures provide a scalable and robust pathway for high-density multimodal tactile integration, with broad applicability in advanced robotic automation and human–robot interaction requiring dexterous and versatile manipulation.

## Supplementary Information

Below is the link to the electronic supplementary material.

Supplementary file1 (DOCX 19485 KB)

Supplementary file2 (MP4 1587 KB)

Supplementary file3 (MP4 1581 KB)
