# Supplementary material for: High-Spatiotemporal and Multimodal Soft Tactile Interface with Layered Architecture for Simultaneous Structural and Thermal Perception
Source: Nanomicro Lett. 2026 Sep 21;19:49. doi: 10.1007/s40820-026-02336-z (PMC13590472; doi:10.1007/s40820-026-02336-z)
Supplement: Supplementary file 1 [file 40820_2026_2336_MOESM1_ESM.docx]

Supporting Information for

**High-Spatiotemporal and Multimodal Soft Tactile Interface with Layered Architecture for Simultaneous Structural and Thermal Perception**

Jaehwan Jang^1, 2, #^, Seong-Min Im^1, 2, #^, Byeong-Sun Park^3^, Jiwon Choi^1^, Dokyung Kim^3^, Min-gu Kim^1, 3, 4,^ ***

^1^ Department of Medical Engineering, College of Medicine, Yonsei University, Seoul 03722, Republic of Korea

^2^ Department of Integrative Medicine, College of Medicine, Yonsei University, Seoul 03722, Republic of Korea

^3^ School of Mechanical Engineering, Yonsei University, Seoul 03722, Republic of Korea

^4^ Graduate School of Medical Science, Brain Korea 21 Project, Yonsei University College of Medicine, Seoul 03722, Republic of Korea

^#^ Jaehwan Jang and Seong-Min Im contributed equally to this work.

* Corresponding author. E-mail: [mgk@yonsei.ac.kr](mailto:mgk@yonsei.ac.kr) (Min-gu Kim)

**Supplementary Text**

**Note S1** **FEM Analysis of the Stacked Soft Capacitive Pressure Sensor**

To investigate the electromechanical characteristics of the capacitive pressure sensor, FEM simulation was performed by comparing the PPC-only and IDC-embedded PPC structures under applied pressure. The simulation was conducted using a multiphysics model coupling the Solid Mechanics and Electrostatics modules to evaluate pressure-induced deformation and the resulting capacitance variation. The FEM model was constructed based on the measured cross-sectional geometry of the actual device. The substrate was modeled with dimensions of 750 μm × 750 μm × 150 μm (width × depth × height), and PDMS was selected from the COMSOL material library. The Ag electrodes were also defined using silver from the COMSOL material library. For the PPC-only structure, the parallel-plate electrode was designed with dimensions of 750 μm × 750 μm × 25 μm. For the IDC-embedded PPC structure, two interdigitated capacitor layers and one parallel-plate capacitor layer were included. The interdigitated electrodes were designed with three fingers length of 450 μm, spacing of 150 μm and width of 150 μm. The dielectric layer of the IDC structure was modeled with dimensions of 750 μm × 750 μm × 35 μm, a Young’s modulus of approximately 70 MPa, and a dielectric constant of 3.8. The microdome layer was designed with a base-film thickness of 15 μm and a dome diameter of 30 μm. The total numbers of mesh elements for the PPC-only and IDC-embedded PPC structures were 328,853 and 123,822, respectively. Fine meshing was applied around the microdome contact regions and Ag electrode areas, where pressure-induced deformation and capacitance variation are dominant.

Before pressure loading, the initial capacitances were calculated to validate the electrical configuration of the FEM model (Fig. S2). The simulated initial capacitances were 371 fF for the PPC-only structure and 880 fF for the IDC-embedded PPC structure, which were in reasonable agreement with the experimental values of 369 fF and 703 fF, respectively. The pressure was then swept from 0 to 1 kPa in 200 Pa increments by applying a boundary load to the top PDMS layer. The Ag electrode region and bottom PDMS layer were fixed, and prescribed displacement conditions were applied to the sidewalls of the microdome-film layer to improve numerical stability during contact deformation. The simulated capacitance variation was mainly governed by deformation of the PPC layer, resulting in similar raw capacitance changes for both structures (Fig. S3). However, the experimentally measured IDC-embedded PPC sensor showed a larger capacitance variation, which may be attributed to deformation of the soft dielectric layer and pressure-induced geometrical changes in the two embedded IDC electrode layers (Fig. S4). These results indicate that the FEM model reasonably captures the dominant PPC-driven pressure response, while additional deformation effects in the embedded IDC layers may contribute to the enhanced capacitance response observed experimentally.

**Note S2** **Fabrication and Performance Comparison of Candidate Temperature Sensors**

Temperature sensors based on MWCNT/PDMS, PEDOT:PSS, and rGO were fabricated using screen printing and spray printing techniques, and their electrical responses to temperature were comparatively evaluated (Fig. S7).

The MWCNT (Graphene Supermarket)/PDMS-based sensor was produced by screen printing a composite ink containing 8 wt% MWCNT dispersed in a PDMS matrix, followed by curing at 100 °C for 2 hours. Despite the use of identical ink formulations, the fabricated devices exhibited considerable variation in their initial resistance values, implying insufficient uniformity in the composite dispersion or printing process. In addition, during temperature sweep tests from 30 °C to 80 °C, the sensors showed a modest resistance change of approximately 11% and demonstrated poor recovery behavior, failing to return to their initial state after cooling.

The PEDOT:PSS (PH1000)-based sensor was fabricated via screen printing following ink viscosity adjustment achieved by stirring at 90–95°C for over 14 hours and O_2_ plasma surface treatment of the substrate, and was subsequently cured at 80°C for 30 minutes. A resistance change of approximately 13% was observed within the 30°C to 60°C range. However, the device exhibited substantial instability in resistance behavior at temperatures exceeding this range, indicating intrinsic thermal limitations of the material system.

In contrast, the rGO-based sensor, fabricated using a spray printing process, demonstrated remarkably superior performance compared to the other materials. During sweep tests from 30°C to 80°C, the rGO sensors exhibited the highest resistance change and excellent linearity, as well as outstanding high-temperature stability. Notably, even in high-temperature environments reaching 130°C, the sensors produced stable and smooth output signals without drifts, suggesting their potential for broad-range temperature sensing applications (Fig. 3).

In summary, while the feasibility of using all three materials for temperature sensing was confirmed, MWCNT/PDMS and PEDOT:PSS require further process optimization their limited recovery and thermal instability. Conversely, rGO demonstrated the most suitable characteristics for temperature sensing applications, ensuring the highest sensitivity and thermal stability among the comparative groups.

**Note S3** **FEM Analysis of the rGO-based Temperature Sensor**

The FEM model of temperature sensor was designed based on the measured cross-sectional geometry of the actual temperature sensor. The simulation was conducted using a multiphysics model coupling the Heat Transfer in Solids and Electric Currents modules to evaluate the temperature distribution and the temperature-dependent resistance variation. The substrate and encapsulation layers were modeled with dimensions of 750 μm × 750 μm × 150 μm (width × depth × height), and PDMS was selected from the COMSOL material library. The electrode and rGO sensing channel were designed with dimensions of 100 μm × 750 μm × 25 μm and 750 μm × 750 μm × 38.5 μm, respectively. To reflect the temperature-dependent electrical response of the electrode and sensing channel, the electrical conductivity of Ag and rGO was defined as a function of temperature using the following equation.

σ_Ag/rGO_ (T) = σ_0_ / (1 + α_Ag/rGO_(T-T_0_)

(σ_0_: Intrinsic electrical conductivity / α: Temperature coefficient of resistance / T, T_0_: Applied, reference temperature)

The intrinsic electrical conductivities of Ag and rGO were set to 61×10^6^ and 60 Sm^-1^, respectively. The temperature coefficients of resistance for Ag and rGO were set to 2×10^-3^ and -5.6×10^-3^K^-1^, respectively. Their thermal conductivities were defined as 429 and 100 Wm^-1^K^-1^, respectively. The mesh consisted of 68,838 elements in total. The mesh consisted of 68,838 elements in total and was finely generated around the electrode and rGO channel regions to analyze the heat-induced conductive response. A heater with dimensions of 750 μm × 750 μm × 150 μm was placed beneath the sensor, and a heat flux boundary condition was applied to provide uniform thermal input to the device. As a result, the FEM simulation reproduced the temperature-dependent response of the actual sensor with a similar decreasing trend in resistance as the temperature increased.

**Supplementary Figures and Tables**

**Fig. S1** Structural characterization of the microdome array.

**Fig. S2** Finite element analysis comparing pressure sensors with and without a capacitor-embedded layer.

**Fig. S3** Electromechanical simulation of capacitive pressure sensors. **a** Simulated electric-field distributions of PPC-only and IDC-embedded PPC structures under pressure. **b** Experimental and simulated capacitance responses as a function of applied pressure.

**Fig. S4** Pressure-induced capacitance response of embedded IDC electrode structures without the PPC sensing layer. Pressure-dependent capacitance variations of the IDC×1 structure without the pressure-sensitive PPC layer were evaluated. The observed capacitance response originated from deformation-induced geometrical changes in the soft dielectric layer and the embedded IDC electrode structure under applied pressure. These results indicate that pressure-induced structural deformation can contribute to capacitance variation even in the absence of the PPC sensing layer.

**Fig. S5** Quantitative sensitivity and hysteresis analysis of the pressure sensor under repeated cycles (*N=50*).

**Fig. S6** Long-term stability of the pressure sensor (0–5 kPa, 100 cycles, 7 days).

**Fig. S7** A Comparison of temperature-dependent electrical responses of MWCNT/PDMS, PEDOT:PSS, and rGO-based temperature sensors.

**Fig. S8** Humidity response of the temperature sensor with and without encapsulation (*N=3*).

**Fig. S9** FEM analysis of the rGO-based temperature sensor. **a** Geometry and functional components of the temperature sensor. **b** Comparison of experimental and simulated resistance responses with increasing temperature. **c** Simulated temperature distribution and heat transfer profiles with increasing temperature.

**Fig. S10** Long-term stability of the temperature sensor (23–35 °C, 100 cycles, 7 days).

**Fig. S11** Effect of combined stimuli on capacitive pressure sensor. Left: a step-like response/recovery to mechanical pressure alone. Right: response/recovery under simultaneous pressure and heat, exhibiting instant change to (i) pressure load/ (iii) unload, with additional effect on signals due to (ii) heat propagation and (iv) dissipation.

**Fig. S12** Temporal response comparison of pressure and temperature sensors under combined pressure–temperature stimulation.

**Fig. S13** Baseline signal variations under different bending curvature radii. Left: pressure sensor. Right: temperature sensor.

**Fig. S14** Sensing performance under different bending curvature radii. Top: capacitive pressure sensor. Bottom: resistive temperature sensor.

**Fig. 15** Stretching-related electromechanical responses of AgNP conductive traces on rigid and deformable substrates under vertically applied pressure.

**Fig. S16** Fabrication process of sensor array using a multilayer interconnection strategy.

**Fig. S17** 3D stacked architecture for via-free multilayer interconnection in array-integrated sensors.

Schematic illustration of the layer-by-layer printed interconnection strategy enabling complex wiring without electrical shorting. The bottom interconnects are first defined via inkjet printing of silver nanoparticle (AgNP) ink on a soft SEBS substrate, followed by the deposition of an insulating layer using insulator ink to electrically isolate crossing nodes. Subsequently, the top interconnects are printed orthogonally on the insulated layer, forming a vertically stacked multilayer wiring structure. This 3D stacked architecture allows for the realization of crossing interconnects without the need for via formation, effectively preventing electrical short circuits between signal lines. Furthermore, this approach significantly reduces wiring complexity and enhances scalability during array expansion, enabling dense integration of sensor arrays with reliable electrical isolation.

**Fig. S18** Photograph of the multi-modal tactile sensor readout system.

**Fig. S19** Batch-to-batch and pixel-to-pixel variation analyses of the 10 × 10 passive-matrix-driven pressure sensor array with the IDC×2 + PPC structure.

**Fig. S20** Batch-to-batch and pixel-to-pixel variation analyses of the 10 × 10 passive-matrix-driven rGO-based temperature sensor array.

**Fig. S21** Crosstalk characterization of the pressure sensor array under localized pressure applied to the center pixels.

**Fig. S22** Crosstalk characterization of the pressure sensor array under localized pressure applied to the left-top corner.

**Fig. S23** Crosstalk characterization of the pressure sensor array under localized pressure applied to the right-top corner.

**Fig. S24** Crosstalk characterization of the pressure sensor array under localized pressure applied to the right-bottom corner.

**Fig. S25** Crosstalk characterization of the pressure sensor array under localized pressure applied to the left-bottom corner.

**Fig. S26** Crosstalk characterization of the temperature sensor array under localized thermal stress applied to the center pixels.

**Fig. S27** Crosstalk characterization of the temperature sensor array under localized thermal stress applied to the left-top corner.

**Fig. S28** Crosstalk characterization of the temperature sensor array under localized thermal stress applied to the right-top corner.

**Fig. S29** Crosstalk characterization of the temperature sensor array under localized thermal stress applied to the right-bottom corner.

**Fig. S30** Crosstalk characterization of the temperature sensor array under localized thermal stress applied to the left-bottom corner.

**Fig. S31** Multimodal tactile recognition of a wrench with curved geometric features.

**Fig. S32** Tactile scanning process.

Schematic illustration of the tactile scanning strategy inspired by the cumulative mapping concept of simultaneous localization and mapping (SLAM). When the target object exceeds the sensing area of the tactile platform, full-field measurement cannot be achieved in a single acquisition. To address this limitation, the target surface is spatially segmented into multiple regions, which are sequentially scanned using the tactile sensor. The acquired pressure and temperature data from each scanned region are then integrated in sequence to reconstruct the overall spatial distribution, enabling complete mapping of object geometry and localized thermal features.

**Fig. S33** Quantitative evaluation of repeatability in tactile scanning for pressure and temperature mapping.

**Table S1** Summary of the performance of printable and stretchable conductors.

NA^✝^: Not Assigned

| **Approach** | **Pressure Sensor** | | | | | | | | | | **Approach** | **Temperature Sensor** | | | | | | | |
| --- | --- | --- | --- | --- | --- | --- | --- | --- | --- | --- | --- | --- | --- | --- | --- | --- | --- | --- | --- |
|  | Material | Spatial Resolution  (Pixel Size) | | Sensitivity  /Sensing Mechanism | | Sensing Range | Response  &Recover | Mechanical Property  /Mechanical Cycling | Ref | |  | Material | Spatial Resolution  (Pixel Size) | TCR  /Sensing Mechanism | | Sensing Range | Response  /Recover | Mechanical Property  /Temperature Cycling | Ref |
| PVD | Au | 4.94 #/cm^2^  (≈3mm) | | 0.047 kPa^-1^  / Capacitive | | 0-550 kPa | 94 & 97 ms | Flexible  / 11,400 | [S1] | | Printing | PEDOT:PSS | 121 #/cm^2^  (≈0.3mm) | -0.09 %/°C  / Thermistor | | 25 - 90 °C | NA^✝^ | Flexible  / NA^✝^ | [S6] |
| PVD | Cu | 44.4 #/cm^2^  (≈0.5mm) | | 0.065 kPa^-1^  / Capacitive | | 0-311 kPa | NA^✝^ | Flexible  / NA^✝^ | [S2] | | Printing | Graphite  +PDMS | 4 #/cm^2^  (≈1.5mm) | 0.0286 %/°C  / Thermistor | | 30 – 110 °C | NA^✝^ | Flexible  / NA^✝^ | [S7] |
| Printing | AgNW | ≈2.56 #/cm^2^  (≈5mm) | | 1.5−104 kPa^-1^  / Capacitive | | 0-40 kPa | 42 & 42 ms | Flexible  / 1,500 | [S3] | | Printing | Pentacene | 17.1 #/cm^2^  (≈2.54mm) | 0.044 %/°C  / Thermistor | | 20 - 100 °C | NA^✝^ | Flexible  / NA^✝^ | [S8] |
| Printing | PEDOT:PSS | ≈31.7 #/cm^2^  (~1 mm) | | 0.56 kPa^-1^  / Capacitive | | -60-20 kPa | NA^✝^ | Soft  / 1,000 | [S4] | | NA | Polyaniline nanofiber | 0.86 #/cm^2^  (≈10mm) | -0.998 %/°C  / NA | | 15 - 45 °C | 1.8 & 3.0 s | Soft  / 11 | [S9] |
| Laser | Conductive Knitted Fabric | 2.33 #/cm^2^  (≈5mm) | | 0.094 kPa^-1^  / Capacitive | | 1-1000 kPa | 140 & 360 ms | Flexible  / 1,000 | [S5] | | Printing | SWCNT/PAA (p-type), SWCNT/PEI (n-type) | 1.14 #/cm^2^  (≈0.1 mm) | NA  / Thermocouple | | 25 - 45 °C | <50 ms & NA^✝^ | Soft  / 9 | [S10] |
| Automated  Printing | AgNP | 46.3 #/cm^2^  (0.75mm) | | 0.021 kPa^-1^  / Capacitive | | 0-325 kPa | 37 & 30 ms | Soft  / 10,000 | This Work | | Automated  Printing | rGO | 46.3 #/cm^2^  (0.75mm) | -0.95 %/°C  /RTD | | 10 - 130 °C | 1.96 & 3.67 s | Soft  /10,000 | This Work |
| **Approach** | **Multimodal Tactile Interface: Integrated Pressure - Temperature Sensor** | | | | | | | | | | | | | | | | | | |
|  | Material | | Spatial Resolution | | Pressure Sensor:  Sensitivity / Sensing Mechanism / Sensing Range / Response & Recover | | | | | Temperature Sensor:  TCR / Sensing Mechanism / Sensing Range / Response & Recover | | | | | Mechanical Property / Mechanical Cycling / Temperature Cycling | | | | Ref |
| Printing | AgNP/rGO | | 4.3 #/cm^2^ | | 0.402 kPa^-1^ / Capacitive / 0-240 kPa / 35 & 18 ms | | | | | -0.72 %/°C / RTD / 30 - 80 °C / 0.32 & 2.51 s | | | | | Flexible / 1,000 / NA^✝^ | | | | [S11] |
| Printing | AgNW | | 4 #/cm^2^ | | NA^✝^ / Strain Sensor / NA^✝^ / NA^✝^ & NA^✝^ | | | | | 10.4 %/°C / Ionic / 20 - 50 °C / NA^✝^ & NA^✝^ | | | | | Soft / 5,000 / 55 | | | | [S12] |
| Printing | AgNP/DPP-DTT | | 28.5 #/cm^2^ | | 2.64 kPa^-1^ / Piezoresistive / 0-20 kPa / NA^✝^ & NA^✝^ | | | | | -2.72 %/°C / TFT based RTD / 25 - 50 °C / NA^✝^ & NA^✝^ | | | | | Flexible / NA^✝^ / NA^✝^ | | | | [S13] |
| PVD+ Printing | Ag/PEDOT:PSS | | Not in Array Format | | 13.29 kPa^-1^ / Piezoresistive / 0-5 kPa / 700 ms & NA^✝^ | | | | | 1.196 %/°C / RTD / 22.4 - 65 °C / 2.4 s & NA^✝^ | | | | | Flexible / 500 / 500 | | | | [S14] |
| PVD+ Printing | Au/PEDOT:PSS+CNT | | 4.5 #/cm^2^ | | 4.11 kPa^-1^ / Capacitive / 0-160 kPa / <100 ms & NA^✝^ | | | | | 0.64 %/°C / Thermistor / 30 - 80 °C / 2.5 s & 4.8 s | | | | | Flexible / 250 / NA^✝^ | | | | [S15] |
| Automated  Printing | AgNP/rGO | | 92.6 #/cm^2^ | | 0.021 kPa^-1^ / Capacitive / 0-325 kPa / 37 & 30 ms | | | | | -0.95 %/°C / RTD / 10 – 130 °C / 1.96 & 3.67 s | | | | | Soft / 10,000 / 10,000 | | | | This Work |

**Supplementary Videos**

**Video S1** Robotic scanning for structural profiles under non-heat conditions.

**Video S2** Robotic scanning for simultaneous structural and thermal profiles.

**Supplementary References**

[S1] M. Farman, R. Prajesh, A. K. Upadhyay, P. Kumar, E. Thouti et al., All-Polydimethylsiloxane-Based Highly Flexible and Stable Capacitive Pressure Sensors with Engineered Interfaces for Conformable Electronic Skin. ACS Appl. Mater. Interfaces **15**(28), 34195–34205 (2023). <https://doi.org/10.1021/acsami.3c04227>
[S2] C. Xu, D. Mei, L. Zhu, Y. Wang, Flexible capacitive pressure sensor array using acoustic-assisted fabrication of microstructures as surface and dielectric layers. Sens. Actuators A Phys. **348**, 114006 (2022). <https://doi.org/10.1016/j.sna.2022.114006>
[S3] Q. Su, C. Liu, T. Xue, Q. Zou, Sensitivity-Photo-Patternable Ionic Pressure Sensor Array with a Wearable Measurement Unit. ACS Appl. Mater. Interfaces **14**(29), 33641–33649 (2022). <https://doi.org/10.1021/acsami.2c09341>
[S4] H. Shi, M. Al-Rubaiai, C. M. Holbrook, J. Miao, T. Pinto et al., Screen-Printed Soft Capacitive Sensors for Spatial Mapping of Both Positive and Negative Pressures. Adv. Funct. Mater. **29**, 1809116 (2019). <https://doi.org/10.1002/adfm.201809116>
[S5] C. Gumus, K. Ozlem, F. Khalilbayli, O. F. Erzurumluoglu, G. Ince et al., Textile-based pressure sensor arrays: A novel scalable manufacturing technique. Micro Nano Eng. **15**, 100140 (2022). <https://doi.org/10.1016/j.mne.2022.100140>
[S6] T. Bücher, R. Huber, C. Eschenbaum, A. Mertens, U. Lemmer et al., Printed temperature sensor array for high-resolution thermal mapping. Sci. Rep. **12**, 14231 (2022). <https://doi.org/10.1038/s41598-022-18321-6>
[S7] W.-P. Shih, L.-C. Tsao, C.-W. Lee, M.-Y. Cheng, C. Chang et al., Flexible Temperature Sensor Array Based on a Graphite-Polydimethylsiloxane Composite. Sensors **10**(4), 3597–3610 (2010). <https://doi.org/10.3390/s100403597>
[S8] X. Ren, K. Pei, B. Peng, Z. Zhang, Z. Wang et al., A Low-Operating-Power and Flexible Active-Matrix Organic-Transistor Temperature-Sensor Array. Adv. Mater. **28**, 4832–4838 (2016). <https://doi.org/10.1002/adma.201600040>
[S9] S. Y. Hong, Y. H. Lee, H. Park, S. W. Jin, Y. R. Jeong et al., Stretchable Active Matrix Temperature Sensor Array of Polyaniline Nanofibers for Electronic Skin. Adv. Mater. **28**, 930–935 (2016). <https://doi.org/10.1002/adma.201504659>
[S10] S. Hwang, D. Jang, H. Kim, J. Kwak, S. Chung, 3D-Printed Soft Temperature Sensors Based on Thermoelectric Effects for Fast Mapping of Localized Temperature Distributions. ACS Appl. Mater. Interfaces. **16**(19), 25071–25079 (2024). <https://doi.org/10.1021/acsami.4c04021>
[S11] S.-M. Im, B.-S. Park, J. Jang, S. Hong, C. Nam et al., Simultaneous in-hand shape and temperature recognition using flexible multilayered sensor arrays for sense-based robot manipulation. Adv. Sens. Res. **4**(7), 70004 (2025). <https://doi.org/10.1002/adsr.70004>
[S12] I. You, D. G. Mackanic, N. Matsuhisa, J. Kang, J. Kwon et al., Artificial multimodal receptors based on ion relaxation dynamics. Science **370**(6519), 961–965 (2020). <https://doi.org/10.1126/science.aba5132>
[S13] Y. Jo, Y. Lee, J. Kwon, S. Kim, G. Ryu et al., 3D active-matrix multimodal sensor arrays for independent detection of pressure and temperature. Sci. Adv. **11**(3), eads4516 (2025). <https://doi.org/10.1126/sciadv.ads4516>
[S14] W. Yang, M. Xie, X. Zhang, X. Sun, C. Zhou et al., Multifunctional Soft Robotic Finger Based on a Nanoscale Flexible Temperature–Pressure Tactile Sensor for Material Recognition. ACS Appl. Mater. Interfaces **13**(46), 55756–55765 (2021). <https://doi.org/10.1021/acsami.1c17923>
[S15] Y. Kumaresan, O. Ozioko, R. Dahiya, Multifunctional Electronic Skin With a Stack of Temperature and Pressure Sensor Arrays. IEEE Sens. J. **21**(23), 26243–26251 (2021). <https://doi.org/10.1109/JSEN.2021.3055458>
